# Quality of life, functional impairment and healthcare experiences of patients with irritable bowel syndrome in Norway: an online survey

**DOI:** 10.1186/s12876-025-03685-6

**Published:** 2025-03-06

**Authors:** Magdy El-Salhy, Mads Johansson, Miriam Klevstul, Jan Gunnar Hatlebakk

**Affiliations:** 1 Department of Clinical Medicine, Department of Gastroenterology, Faculty of Medicine and Dentistry, University of Bergen, Bergen, Norway; 2 National Centre for Functional Gastrointestinal Disorders, Department of Medicine, Haukeland University Hospital, Bergen, Norway; 3 Norwegian Gastrointestinal Association, Postboks 808 Sentrum, Oslo, 0104 Norway

**Keywords:** Embarrassment, Healthcare experiences, Loss of freedom, Sham, Unemployment

## Abstract

**Background:**

The present study is an online survey to determine the quality of life and functional impairment caused by irritable bowel syndrome (IBS) and the healthcare experiences of IBS patients in Norway, which has not been done previously.

**Methods:**

An author-developed, online questionnaire using Microsoft Forms program was applied. The questionnaire comprised 52 questions. The questionnaire was posted from 1 March to 17 April (a 48-day period) on the Norwegian Gastrointestinal Association website and in its magazine and social media posts.

**Results:**

The questionnaire was completed by 2727 patients. Of these patients 327 were excluded because they have not been diagnosed by a medical doctor. IBS reduced the quality of life in 97% of affected patients, including the social life in 90% and the sexual life in 69%. The unemployment rate of IBS patients in Norway 38%, which is 10-fold higher than that of the general population, and 94% of IBS patients reported that IBS impaired their work/study performance. About half of the patients had to discuss their abdominal symptoms with their general practitioner (GP) more than 10 times and wait more than 1 years before being diagnosed. Only 34% and 48% were satisfied with the help they received from their GP and gastroenterologist, respectively. Only 18% and 43% of the IBS patients felt that their GP and gastroenterologist, respectively, were sufficiently knowledgeable about IBS. Only 21% of the IBS patients was satisfied with the treatment they received.

**Conclusions:**

IBS markedly reduces the affected patient’s quality of life and their working productivity. IBS patients are generally dissatisfied with the clinical management they receive from GPs and gastroenterologists.

**Supplementary Information:**

The online version contains supplementary material available at 10.1186/s12876-025-03685-6.

## Background

Irritable bowel syndrome (IBS) is a common gut–brain interaction disorder with a prevalence of 10% worldwide [1]. The prevalence of IBS is higher in females than males, and highest in adults younger than 40 years [1]. IBS markedly reduces the quality of life of affected patients, and it also represents an economic burden to society [1]. The annual healthcare costs for IBS patients have been estimated at £45.6–200 million in the UK, US$ 2 billion in China and €3–4 billion in Germany [1]. Moreover, indirect costs to society from lost work productivity and sickness or disability benefits are considerable. The annual indirect costs for IBS across 13 European countries have been estimated to be €2,314 per person [1]. Furthermore, stress levels are significantly higher in the partners of IBS patients than in those of healthy subjects [1]. The societal economic costs of distress to family members of IBS patients are unknown, but they could be considerable [1].

The prevalence of IBS in the Norwegian population increased from 7.5% during 2006–2008 to 9.5% during 2017–2019 [2]. Both physical and mental aspects of the quality of life of Norwegian IBS patients have been reported to be significantly impaired [3]. However, the functional impairment caused by IBS and the healthcare experiences of IBS patients in Norway have not been investigated previously. The present study performed an online survey to determine the impact of IBS on the quality of life, functional impairment and healthcare experiences of patients in Norway.

## Methods

An author-developed, online questionnaire using Microsoft Forms program was applied (Supplementary material 1). The questionnaire was examined by The Norwegian National Centre for Functional Gastrointestinal Disorders to ensure its quality. The questionnaire comprised 52 questions: 6 questions on demographic data, 9 on the quality of life and functional impairment, and 37 on the healthcare experiences of patients. The questionnaire was posted and open for the general public from 1 March to 17 April 2023 (a 48-day period) on the Norwegian Gastrointestinal Association website and in its magazine and social media posts. The survey was anonymous.

### Ethics

An application for the study was submitted to the West Regional Committee for Medical and Health Research Ethics, Bergen, Norway (Number 736660). The Committee answer was that “Research using anonymous survey data is not within the scope of the Health Research Act § 11, and can therefore be done without approval from the Regional Committee for Medical and Health Research Ethics or informed consent to participate.”

## Results

The questionnaire was completed by 2727 patients. Of these patients 327 were excluded because they have not been diagnosed by a medical doctor, or under 18 years of age. The detailed survey answers are given in the supplementary material 1. The demographic and clinical characteristics of the respondents are summarized in Table 1.

**Table 1:** Demographic and clinical characteristics of the irritable bowel syndrome (IBS) patients who participated in the survey

Number of participants	2727
Age, years	40 [18–85]
Sex, female/male	2430/297
Residence in Ostlandet	1269 (47%)
Residence in Vestlandet	626 (23%)
Residence in Sørlandet	168 (6%)
Residence in Midt-Norge	369 (13%)
Residence in Nord-Norge	286 (11%)
Residence abroad	9 (< 1%)
Diarrhoea	1024 (37%)
Constipation	728 (27%)
Both diarrhoea and constipation	975 (36%)
Duration of IBS, years	10 [1–30]
Abdominal pain	2299 (84%)
Bloating	2438 (89%)
Headache	1153 (42%)
Fatigue	2673 (98%)
Employed	1437(53%)
Student	255 (9%)
Unemployed	1035 (38%)

Data are *n*, *n* (%), or median [range] values

### Quality of life and functional impairment

**Fig. 1:**
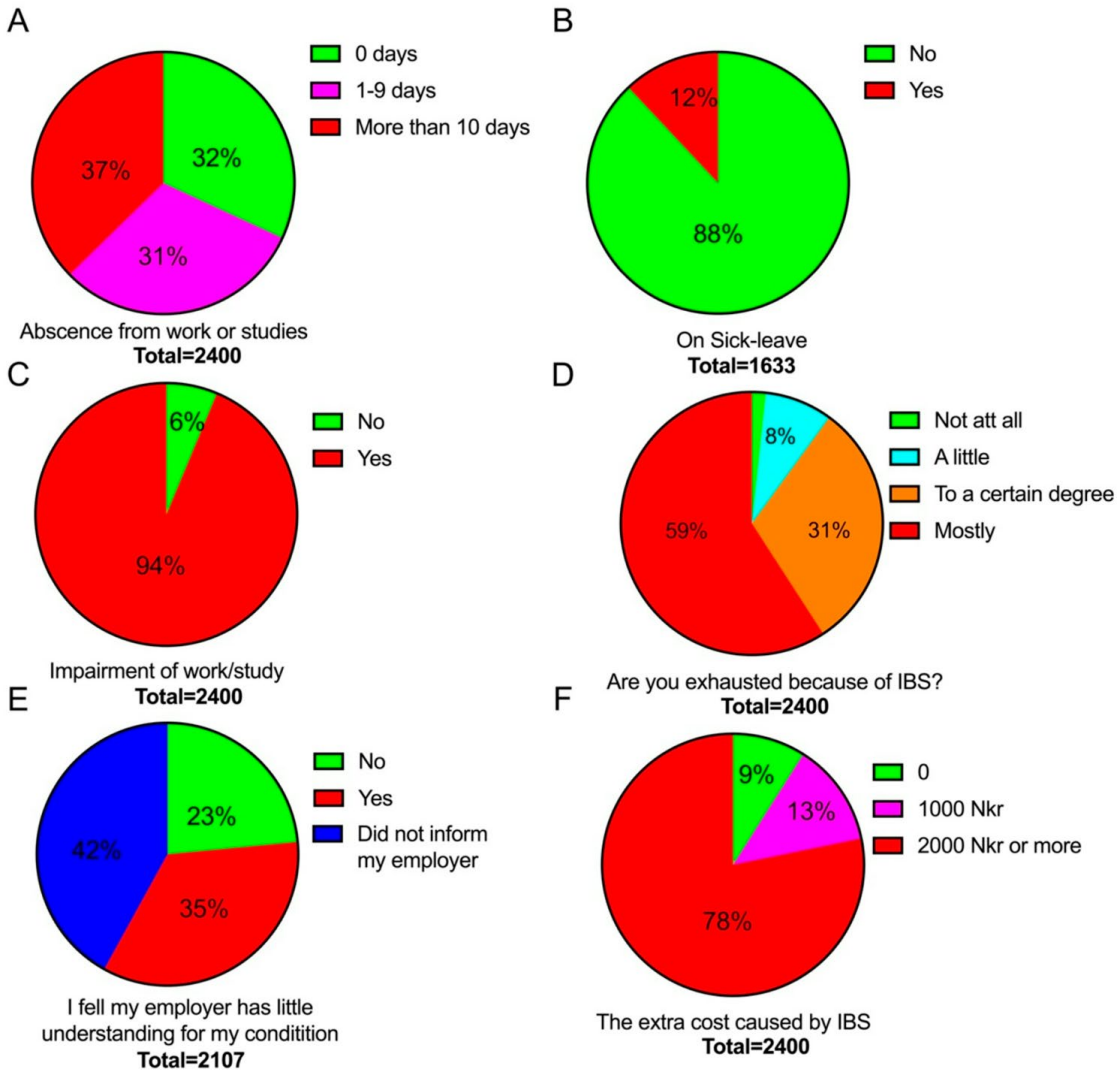
Effects of irritable bowel syndrome (IBS) on work/study and on personal finances: absenteeism from work/studies (**A**), sick leave (**B**), being affected by fatigue (**C**), impairment of work/study (**D**), employer understanding of the disability caused by IBS (**E**) and economic burden on patients (**F**)

Absences from work or study for more than 10 days annually were reported by 37% of the IBS patients (Fig. 1A), 12% were on sick leave because of IBS (Fig. 1B), 94% reported that IBS impaired their work/study (Fig. 1C), 98% experienced fatigue caused by IBS (Fig. 1D), 35% felt that their employer did not understand their condition and 42% did not informed their employer that they had IBS (Fig. 1E), and 91% considered IBS to be an economic burden (Fig. 1F).

**Fig. 2:**
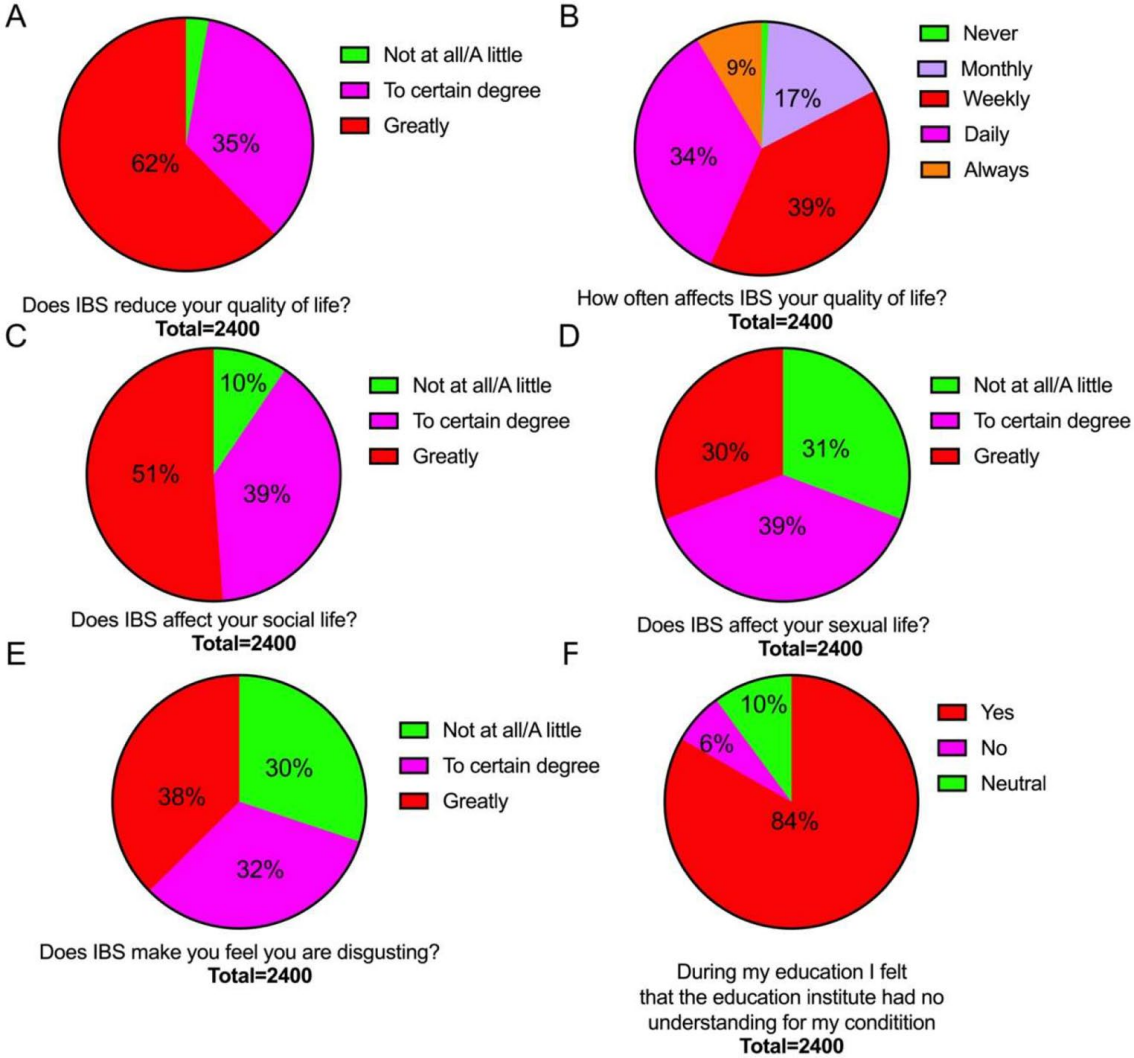
Reduction in quality of life (**A**), frequency of such reduction (**B**), and IBS effects on social life (**C**), sexual life (**D**), feeling disgusting (**E**) and understanding of the education institute for the patients’ condition (**F**)

IBS reduced the quality of life in 97% of the IBS patients, markedly so in 62% of them (Fig. 2A). About 73% reported that they experienced a reduction in their quality of life on a daily basis/always (Fig. 2B). IBS affected the social and sexual lives of 90% and 69% of them, respectively (Fig. 2C and D). IBS made 70% of the patients feel disgusting (Fig. 2E) and 84% felt during their education institute had no understanding of their condition (Fig. 2F).

### Patients’ healthcare experiences

**Fig. 3:**
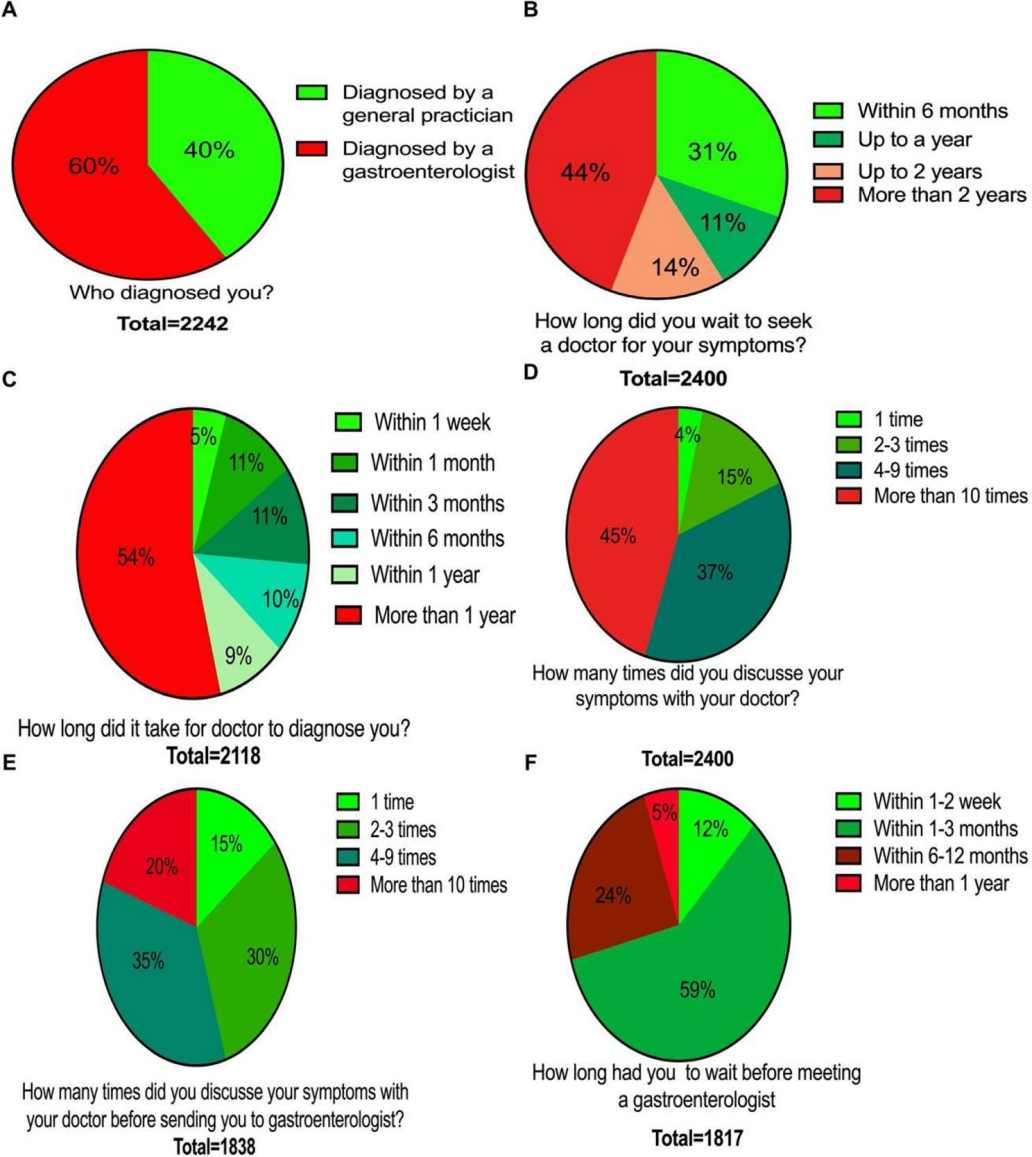
Person who performed the diagnosis (**A**), duration before seeking a doctor (**B**), duration to reach the diagnosis (**C**), number of times that patients had to discuss their problems with their general practitioner (GP) before reaching a diagnosis (**D**), number of times that patients had to discuss their problems with their GP before being referred to a gastroenterologist (**E**) and how long they waited before seeing a gastroenterologist (**F**)

**Fig. 4:**
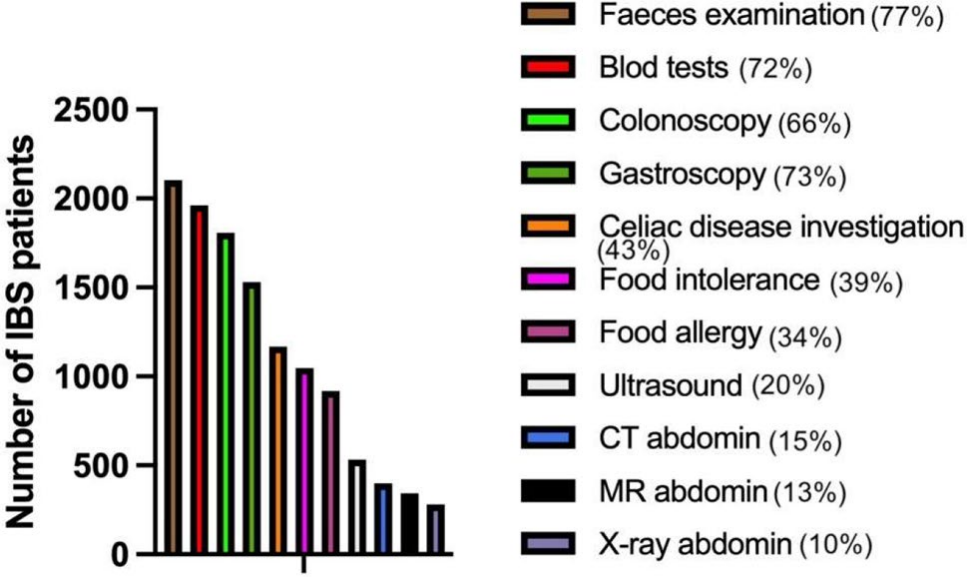
The different tests used to investigate IBS patients before reaching a diagnosis. CT, computed tomography; MRI, magnetic resonance imaging

Only 40% of the patients were diagnosed by a general practitioner (GP) and the other 60% by a gastroenterologist (Fig. 3A). About half of the patients (44%) waited more than 2 years before seeking a doctor for help with their IBS (Fig. 3B). Only 4% of the patients had to discuss their abdominal symptoms with their GP once and 45% of the patients had to drift their symptoms more than 10 times and 54% waited more than 1 year before a diagnosis was reached (Fig. 3C and D). About half of the patients (45%) needed to discuss their abdominal times symptoms 1–3 times before being referred to a gastroenterologist, with the remainder having to describe their symptoms from 4 to more than 10 times before being referred to a gastroenterology (Fig. 3E). On the other hand, only a similarly small proportion (29%) had to wait more than 3 months before seeing a gastroenterologist (Fig. 3F). The different tests used to investigate the IBS patients are shown in Fig. 4.

**Fig. 5:**
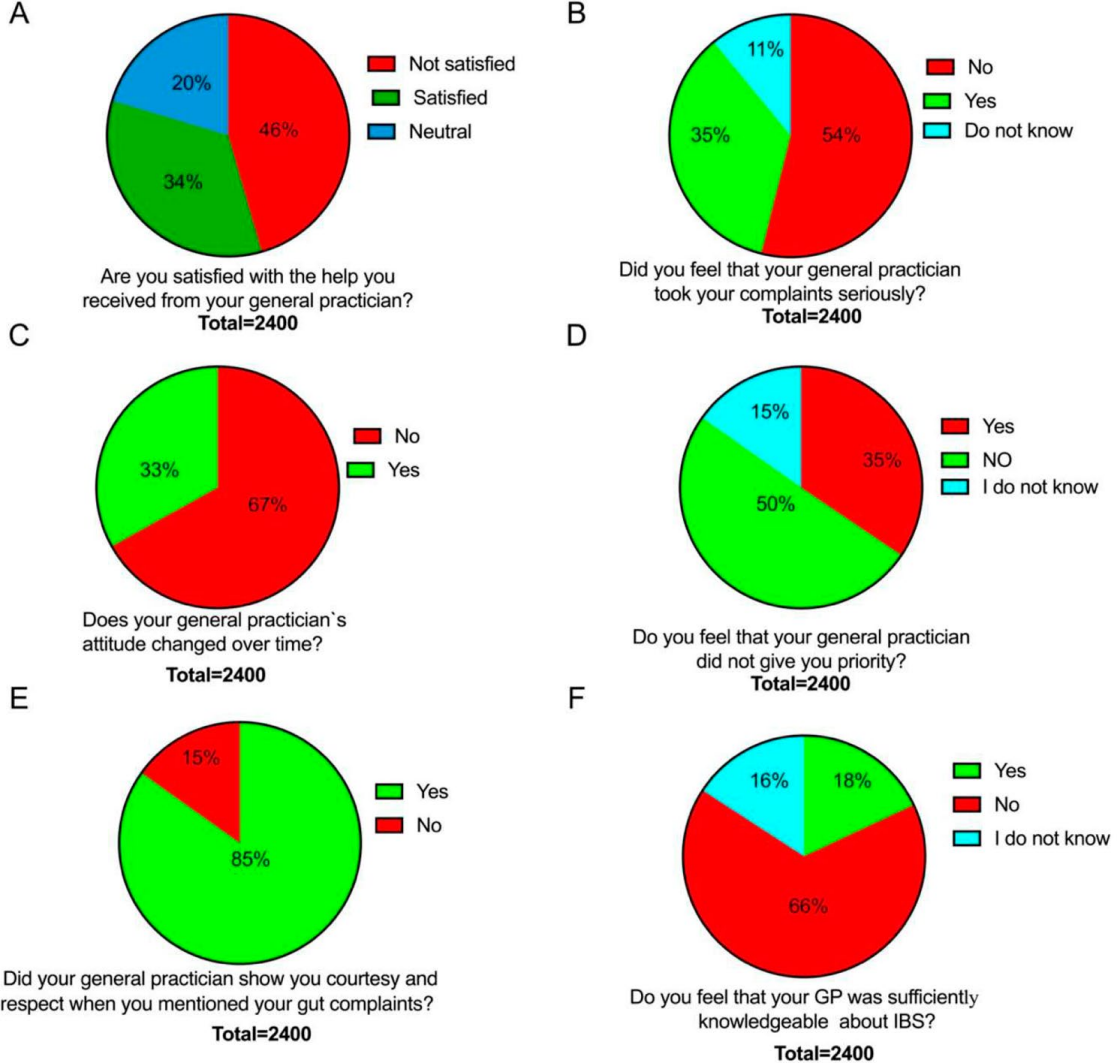
Experiences of IBS patients with their GP. Satisfaction with the help they received (**A**), whether the GP took their complaints seriously (**B**), whether the attitude of their GP changed to the better over time (**C**), whether their GP gave them priority (**D**), whether their GP showed them respect and courtesy (**E**) and whether they considered that their GP was sufficiently knowledgeable about IBS (**F**)

Only 34% of the IBS patients were satisfied with the help they received from their GP (Fig. 5A), with only 35% reporting that their GP took their complaints seriously (Fig. 5B) and 33% reporting that the attitude of their GP changed to the better over time (Fig. 5C). Only 35% of IBS patients experienced that their GP did not give them priority (Fig. 5D), while 85% reported that their GP showed them respect and courtesy (Fig. 5E). Only 18% of the IBS patients felt that their GP was sufficiently knowledgeable about IBS (Fig. 5F).

**Fig. 6:**
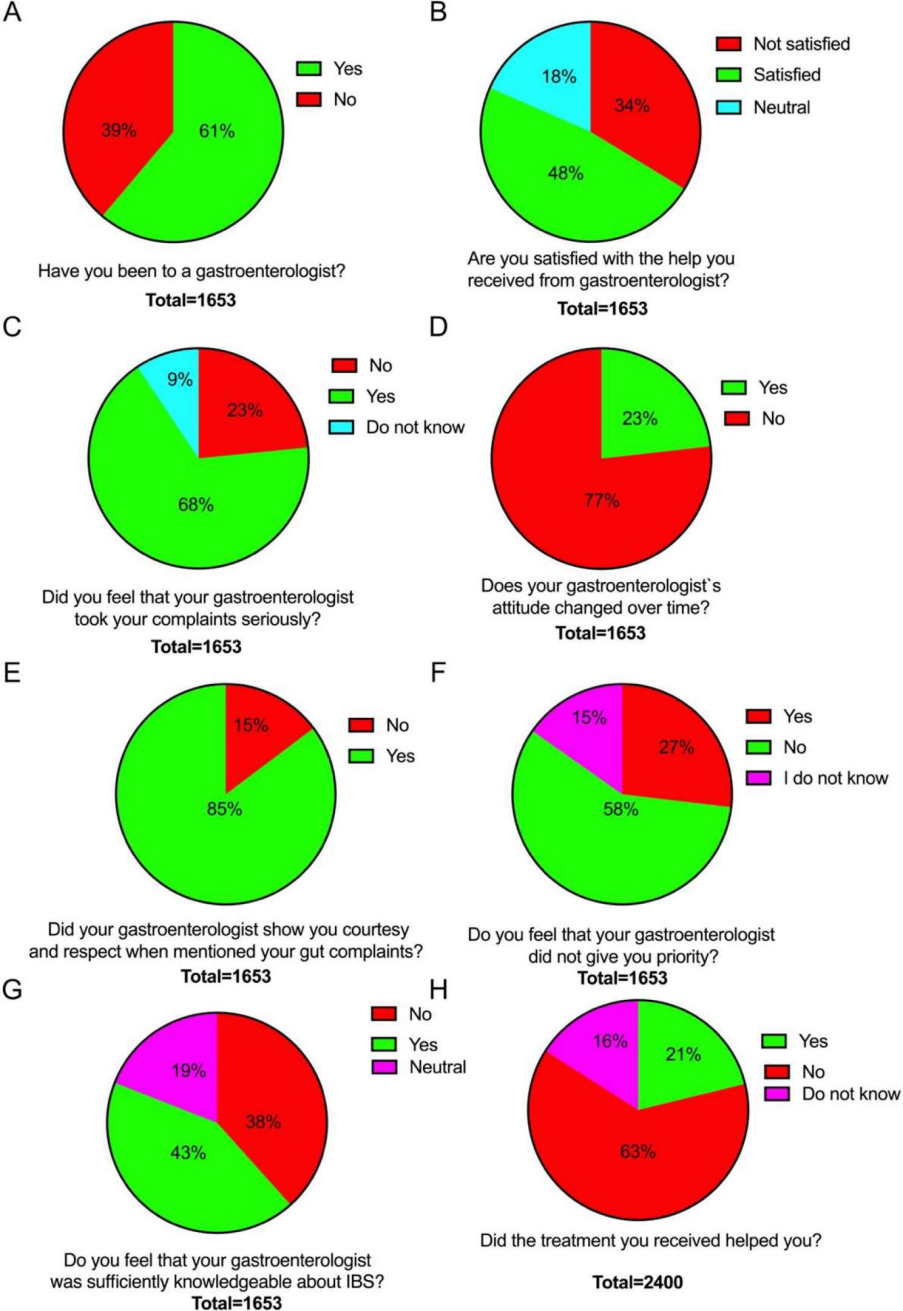
Experiences of IBS patients with their gastroenterologist. Been evaluated by a gastroenterologist (**A**), satisfaction with the help they received (**B**), whether the gastroenterologist took their complaints seriously (**C**), whether their attitude of their gastroenterologist changed to the better over time (**D**), whether their gastroenterologist showed them respect and courtesy (**E**), whether their gastroenterologist gave them priority (**F**), whether they considered that their gastroenterologist was sufficiently knowledgeable about IBS (**G**) and whether the treatment actually helped them (**H**)

More than 60% of the IBS patients had seen a gastroenterologist (Fig. 6A), and 48% were satisfied with the help they received (Fig. 6B). Only 23% reported that they felt that the gastroenterologist did not take their complaints seriously (Fig. 6C), A and 23% reported that the attitude of the gastroenterologist changed to the better over time (Fig. 6D). Large proportion of the patients (85%) reported that the gastroenterologist showed them respect and courtesy (Fig. 6E), and 27% experienced that they had not been given priority (Fig. 6F). Only 43% of the patients believed that their gastroenterologist was sufficiently knowledgeable about IBS (Fig. 6G). Only 21% of the patients reported that the treatment they received from either their GP or gastroenterologist actually helped them (Fig. 6H).

## Discussion

IBS significantly reduces the quality of life of affected patients in the Western world (specifically, in North America and Europe) [3–6]. IBS adversely affects daily functioning, thoughts, feelings and behaviours [7]. Feelings of the loss of freedom, spontaneity and social contacts, and of fearfulness, shame and embarrassment have been reported by patients with IBS [1, 7]. Furthermore, IBS symptoms impair earnings and the abilities to travel and have a normal sex life [1, 7, 8]. The quality of life of patients with IBS is reduced by similar degrees as for other chronic diseases such as inflammatory bowel disease, rheumatoid arthritis and kidney failure, and is reduced by greater degrees than for patients with diabetes mellitus, asthma and gastroesophageal reflux [4, 6, 9, 10]. Almost all (97%) of the patients in the present study reported that IBS reduces their quality of life. Of these patients, 73% reported that it affected their quality of life markedly, and on a daily basis in about 34% of them. Most of the included patients reported that IBS impaired their social life (90%), sexual life (69%) and work/study (94%). Moreover, 70% of the patients reported that they felt disgusting. IBS is also an economic burden to the patients due to the extra costs associated with the condition (91%), and the loss of earnings from unemployment (29%) or work absences (12%). Only 23% of the patients reported that their employers understood their condition, with only 6% stating that during their studies the educational institute demonstrated an understanding of their condition. It is noteworthy that 42% of the patients with IBS did not inform their employer about their condition, which could be due to the fear of being stigmatized by their employer and co-workers or even losing their job. Together these findings imply that IBS exerts marked adverse effects on these patients’ quality of life.

In addition to IBS symptoms, 90% of the patients who completed the questionnaire suffered from chronic fatigue. It has also been reported previously that chronic fatigue is associated with IBS in up to 96% of patients with IBS, and it was suggested that IBS and chronic fatigue have the same underlying mechanisms [11, 12]. Associations of 11 intestinal bacteria with both IBS and chronic fatigue were reported very recently, further supporting the assumption of a common pathophysiology [13].

IBS patients constitute a considerable workload for both the primary and secondary healthcare systems, comprising 12–14% of primary-care patient visits and 28% of referrals to gastroenterologists [14–16]. IBS as a reason to visit to a physician is more common than for diabetes, asthma and hypertension [17, 18]. There are substantial healthcare costs for resource use related to healthcare delivery, condition investigations, hospitalization, visits to casualty departments and to emergency rooms and treatments [1]. For example, the annual healthcare costs for IBS patients have been estimated at £45.6–200 million in the UK, €3–4 billion in Germany, €43 million in Finland and US$ 2 billion in China). In the USA the healthcare costs have been estimated at US$ 1,562–7,547 per patient annually [19–25]. In the present survey, 37% of the patients with IBS had to visit their GP more than 3 times before being diagnosed, and 45% more than 10 times. Moreover, investigating their condition required a large number of blood tests, faecal pathological bacterial cultivations, endoscopies, radiological examinations and ultrasound examinations. All of these factors contributed to the substantial healthcare costs.

The uunemployment rate in Norway in December 2022 was 3.4% (https://www.ceicdata.com/en/indicator/norway/unemployment-rate). The present survey revealed that the unemployment rate among IBS patients was 38%, which is therefore more than 10-fold that of the general population. Moreover, this study showed that 12% of IBS patients were on sick leave and 37% were absent from work/study for more than 10 days annually. In addition, 94% of the IBS participating patients reported that IBS impaired their work/study. These aspects together indicate that the indirect costs to society in the form of lost work productivity and sickness or disability benefits are considerable.

Many patients with IBS are dissatisfied with the clinical management they receive [1, 26]. The present survey found that 44% of the IBS patients waited more than 2 years before seeking a doctor for their symptoms, and that about half of them had to discuss their symptoms with their GP more than 10 times and 54% waited more than 1 year before being diagnosed. Most of the IBS patients (85%) reported that both GPs and gastroenterologists showed them courtesy and respect when discussing their symptoms, but a considerable proportion of them did not feel that their GP (54%) or gastroenterologist (23%) took their complaints seriously. It noteworthy that it takes time to gain the indispensable patients confidence where MD’s time for each patient is limited. A considerable proportion of the IBS patients reported that they were not satisfied with the help they received from their GP (46%) or gastroenterologist (34%). Only 21% reported that the treatment they received actually helped them. It may therefore be concluded that Norwegian IBS patients, like other IBS patients worldwide, are dissatisfied with the clinical management that is currently applied for their condition. It is worthy of note that 60% of the IBS patients participated in the survey was diagnosed by a gastroenterologist though the guidelines in Norway recommends that IBS patients should be diagnosed in primary care, and that referral to a gastroenterologists should be limited to patients with special features or heavy symptom burden.

Only 18% and 43% of the IBS patients who participated in this survey believed that their GP and gastroenterologist, respectively, were sufficiently knowledgeable about IBS. IBS is diagnosed based on symptom assessments as described by the Rome criteria [27–33]. The Rome criteria without red flags and medical history and physical examinations are effective in diagnosing IBS. The effectiveness of the Rome criteria was confirmed in a study of about 1,500 Norwegian IBS patients [34]. Despite this established diagnostic method, GPs and gastroenterologists subjecting the survey participants to a unnecessary large number of tests including endoscopy of the upper and lower gastroenterology tracts. The findings in this survey showed that there is a need for physicians who care for this patient group to improve insight and develop communicative skills, which could save resources and suffering.

The main strength of this survey was that it was answered by a relatively large number of IBS patients. However, the main limitation of this study, like other online survey, was that it was answered by people used to digital habits. Moreover, 327 who answered the questionnaire were excluded because they were not diagnosed by a medical doctor. These excluded persons could have IBS, but did not seek medical help.

## Conclusions

IBS reduces greatly the affected patient’s quality of life and their working productivity. IBS causes substantial healthcare costs for resource use related to healthcare delivery such as condition investigations, hospitalization, visits to casualty departments and to emergency rooms and treatments. Furthermore, the indirect costs of IBS to the society in form of lost work productivity or disability benefits are considerable. IBS patients are generally dissatisfied with the clinical management they receive from GPs and gastroenterologists and the only a minority of them believes that GPs and gastroenterologists were sufficiently knowledgeable about IBS.

## Electronic supplementary material

Below is the link to the electronic supplementary material.

Supplementary Material 1

## Data Availability

Data presented in this study are available from the corresponding author on request.
