# Supplementary material for: Quality of life, functional impairment and healthcare experiences of patients with irritable bowel syndrome in Norway: an online survey
Source: BMC Gastroenterol. 2025 Mar 6;25:143. doi: 10.1186/s12876-025-03685-6 (PMC11883911; doi:10.1186/s12876-025-03685-6)
Supplement: Supplementary file 1 — Supplementary Material 1 [file 12876_2025_3685_MOESM1_ESM.docx]

**Supplementary Material**

The questionnaire used was in Norwegian

**English translation**

Survey on Living with Irritable Bowel Syndrome (IBS)

The Norwegian Gastrointestinal Association would like to get a better understanding of how is to have and to live with irritable bowel syndrome (IBS). Accordingly, we are carrying out this survey. We shall use the results of this survey to increase the awareness of the society of the problems faced by IBS patients. We would appreciate it if you answer the survey. The survey has around 50 questions and takes about 10 minutes. The survey is anonymous.

1. Sex


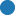
 Male


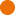
 Female

1. Age

| 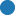 Under 13 |  |
| --- | --- |
| 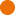 13-17 years |  |
| 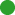 18-24 years |  |
| 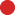 25-29 years |  |
| 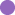 30-34 years |  |
| 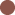 35-39 years |  |
| 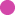 40-44 years |  |
| 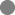 45-49 years |  |
| 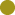 50-54 years |  |
| 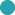 55-59 years |  |
| 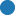 60-64 years |  |
| 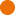 65-69 years |  |
| 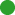 70-74 years |  |
| 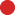 75-79 years |  |
| 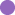 80-84 years |  |
| 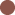 85-89 years |  |
| 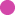 Over 90 |  |

1. Place of abode

| 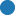 Eastern Norway |  |
| --- | --- |
| 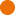 Western Norway |  |
| 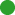 Southern Norway |  |
| 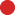 Central Norway |  |
| 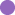 Northern Norway |  |
| 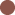 I don't live in Norway |  |
|  |  |

1. Employment status

| 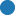 Employed |  |
| --- | --- |
| 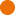 Student |  |
| 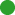 Incapacitated (100%) |  |
| 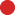 Partially incapacitated |  |
| 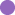 On sick leave (100%) |  |
| 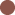 Partially on sick leave |  |
| 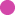 Old age pensioner |  |
| 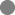 School pupil |  |
| 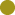 Homemaker |  |
| 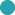 Receiving a work assessment allowance |  |
| 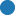 Work placement |  |
| 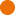 Unemployed |  |
| 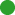 Laid off from work |  |
| 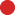 Other |  |
|  |  |

1. Estimate the average number of days per year that you are/have been absent from work or education due to your irritable bowel.

| 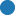 0 days |  |
| --- | --- |
| 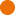 1-9 days |  |
| 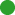 10-19 days |  |
| 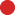 20-29 days |  |
| 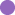 30-39 days |  |
| 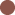 40-49 days |  |
| 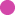 50-59 days |  |
| 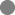 60-69 days |  |
| 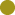 70-79 days |  |
| 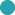 80-89 days |  |
| 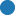 3-4 months |  |
| 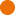 5-6 months |  |
| 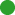 7-8 months |  |
| 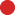 9-10 months |  |
| 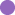 11-12 months |  |
|  |  |

1. Are you incapacitated, on sick leave or receiving a work assessment allowance because of your irritable bowel?


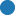
 Yes, IBS is the main reason I am incapacitated, on sick leave or receiving a work assessment allowance


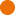
 Yes, IBS is one of the reasons why I am incapacitated, on sick leave or receiving a work assessment allowance


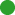
 No

1. Have you been diagnosed with irritable bowel syndrome?

| 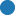 Yes, it was diagnosed by my GP. |  |
| --- | --- |
| 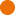 Yes, it was diagnosed by a gastrointestinal specialist. |  |
| 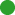 Yes, it was diagnosed by another doctor (not a GP or gastrointestinal specialist). |  |
| 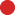 No, I haven't received a diagnosis from the health service, I arrived at this conclusion on my own. |  |
| 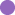 I don't have irritable bowel syndrome. |  |
| 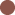 Other |  |

1. What irritable bowel symptoms do you have?

| 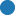 Diarrhoea |  |
| --- | --- |
| 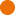 Constipation |  |
| 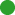 Stomach pain |  |
| 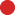 Bloated stomach |  |
| 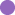 Headache |  |
| 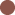 Chronic fatigue |  |
| 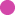 Other |  |
|  |  |

1. Approximately how long ago is it since you first developed symptoms of irritable bowel syndrome?

| 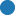 Less than six months ago |  |
| --- | --- |
| 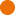 6-12 months |  |
| 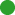 1-2 years |  |
| 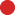 3-5 years |  |
| 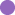 6-10 years |  |
| 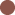 11-14 years |  |
| 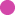 15-19 years |  |
| 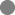 20-24 years |  |
| 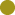 25-29 years |  |
| 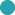 Over 30 years ago |  |
|  |  |

1. If a doctor has diagnosed you with irritable bowel syndrome, approximately how long ago did you receive the diagnosis?

| 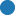 Less than six months ago |  |
| --- | --- |
| 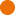 6-12 months |  |
| 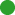 1-2 years |  |
| 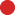 3-5 years |  |
| 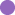 6-10 years |  |
| 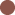 11-14 years |  |
| 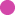 15-19 years |  |
| 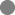 20-24 years |  |
| 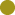 25-29 years |  |
| 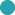 Over 30 years ago |  |
| 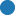 I haven't been diagnosed by a doctor |  |

1. To what extent does irritable bowel syndrome impair your quality of life?


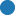
 Not at all


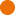
 To a small degree


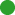
 To some extent


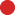
 To a great extent


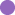
 To a very great extent

1. How often do you feel your quality of life is reduced because of your irritable bowel?


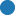
 Never


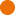
 Monthly


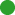
 Weekly


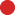
 Daily

Always

1. To what extent does irritable bowel syndrome impair your work/studies?

Not at all

To a small degree

To some extent

To a great extent

To a very great extent

1. To what extent does irritable bowel syndrome impair your social life?

Not at all

To a small degree

To some extent

To a great extent

To a very great extent

1. To what extent does irritable bowel syndrome impair your sex life?

Not at all

To a small degree

To some extent

To a great extent

To a very great extent

1. Are you often tired because of your irritable bowel?

Not at all

To a small degree

To some extent

To a great extent

To a very great extent

1. Do you feel repulsed by your irritable bowel?

Not at all

To a small degree

To some extent

To a great extent

To a very great extent

1. Approximately how long did it take before you visited a doctor after the onset of the first symptoms of irritable bowel syndrome?

| - A couple of days |  |
| --- | --- |
| - 1 week |  |
| 2-4 weeks |  |
| 1-3 months |  |
| 4-6 months |  |
| 6-12 months |  |
| 1-2 years |  |
| 2-3 years |  |
| 4-5 years |  |
| More than 5 years |  |
| I haven't seen a doctor because of this. |  |
| Don’t know |  |

1. Approximately how many times have you discussed your intestinal problems with your GP?

| Once |  |
| --- | --- |
| 2-3 times |  |
| 4-5 times |  |
| 6-9 times |  |
| 10-14 times |  |
| 15-19 times |  |
| 20-49 times |  |
| 50 times or more |  |
| I’ve never mentioned my intestinal problems to my GP. |  |
| Don’t know |  |

1. Approximately how many times did you discuss your intestinal problems with your GP before he/she sent you to a specialist?

| - Once |  |
| --- | --- |
| - 2-3 times |  |
| 4-5 times |  |
| 6-9 times |  |
| 10-14 times |  |
| 15-29 times |  |
| 30 times or more |  |
| I’ve never mentioned my intestinal problems to my GP. |  |
| My GP has never sent me to a specialist. |  |
| Don’t know |  |

1. Have you had to change your GP to be referred to a medical specialist?

Yes

No

1. How long did it take before you saw a specialist after your GP sent a referral?

| 1 day |  |
| --- | --- |
| 2-3 days |  |
| Approx. one week |  |
| Approx. 14 days |  |
| Approx. one month |  |
| 2-3 months |  |
| 4-6 months |  |
| 7-12 months |  |
| More than one year |  |
| More than two years |  |
| Don’t know |  |
| My GP has never sent me to a specialist. |  |

1. Have you seen a private specialist at your own expense for your intestinal problems?

Yes

No

1. Overall, approximately how long did it take from the time when you first saw a doctor until you were diagnosed with IBS?

| - Less than one week |  |
| --- | --- |
| - 1-3 weeks |  |
| Approx. one month |  |
| 2-3 months |  |
| 4-6 months |  |
| 7-12 months |  |
| 1-2 years |  |
| 3-4 years |  |
| 5 years or more |  |
| I haven't received a diagnosis from the health service, I arrived at this conclusion on my own. |  |
| I haven't spoken to a doctor about my intestinal problems. |  |
| Don’t know |  |

1. What tests and examinations were carried out when investigating irritable bowel syndrome? (you can select more than one option)

| Gastroscopy |  |
| --- | --- |
| - Colonoscopy |  |
| Blood tests |  |
| Stool samples |  |
| MRI |  |
| X-ray |  |
| Ultrasound |  |
| CT |  |
| Food allergy test |  |
| Food intolerance tests |  |
| Investigation for celiac disease |  |
| Don’t know |  |
| No tests have been taken |  |
| Other |  |

1. How satisfied are you with the help you have received from the GP service for your irritable bowel?

Very dissatisfied

Dissatisfied

Slightly dissatisfied

Neither dissatisfied nor satisfied

Somewhat satisfied

Satisfied

Very satisfied

1. Have you experienced your GP not believing you when you’ve told him/her about your stomach problems?

Yes

No

Don’t know

1. In all encounters with your GP, how often have you experienced that he/she has not believed you when you’ve told him/her about your stomach problems?

| - Never |  |
| --- | --- |
| - Very rarely |  |
| Rarely |  |
| Often |  |
| Very often |  |
| Always |  |

1. Has this experience changed since the first time you told your GP about your stomach problems?

Yes, I find that my GP believes me more now than he/or she did during the first consultation.

Yes, I find that my GP believes me less now than during the first consultation.

No, I find that my GP is still the same as during the first consultation.

I haven't spoken to my GP about my stomach problems.

1. Have you experienced that your GP hasn't prioritised helping you when you've told him/her about your stomach problems?

Yes

No

Don’t know

1. In all encounters with your GP, how often have you experienced that he/she has prioritised helping you when you’ve told him/her about your stomach problems?

| Never |  |
| --- | --- |
| Very rarely |  |
| Rarely |  |
| Often |  |
| Very often |  |
| Always |  |

1. Has this experience changed since the first time you told your GP about your stomach problems?

Yes, I find that my GP prioritises helping me more today than during the first consultation.

Yes, I find that my GP prioritises helping me less today than during the first consultation.

No, I find that my GP is still the same as during the first consultation.

I haven't spoken to my GP about my stomach problems.

1. How often do you find that your GP meets you with courtesy and respect when you tell him/her about your stomach problems?

| Never |  |
| --- | --- |
| Very rarely |  |
| Rarely |  |
| Often |  |
| Very often |  |
| Always |  |

1. To what extent do you agree or disagree with the following statement:

I find that the GP service has sufficient expertise on irritable bowel syndrome.

| - Totally disagree |  |
| --- | --- |
| - Disagree |  |
| Slightly disagree |  |
| Neither disagree nor agree |  |
| Slightly agree |  |
| Agree |  |
| Totally agree |  |

1. Have you been in contact with the specialist health service for your stomach problems?

Yes

No

1. How satisfied are you with the help you have received from the specialist health service for your irritable bowel?

Very dissatisfied

Dissatisfied

Slightly dissatisfied

Neither dissatisfied nor satisfied

Somewhat satisfied

Satisfied

Very satisfied

1. Have you experienced that health professionals in the specialist health service have not believed you when you’ve told them about your stomach problems?

Yes

No

Don’t know

1. In all your encounters with the specialist health service, how often have you experienced that health professionals have not believed you when you’ve told them about your stomach problems?

| - Never |  |
| --- | --- |
| - Very rarely |  |
| Rarely |  |
| Often |  |
| Very often |  |
| Always |  |

1. Has this experience changed since the first time you told health professionals in the specialist health service about your stomach problems?

Yes, I find that health professionals in the specialist health service believe me more today than during the first consultation.

Yes, I find that health professionals in the specialist health service believe me less today than during the first consultation.

No, I have the same experience of health professionals in the specialist health service today as during the first consultation.

1. Have you experienced that health professionals in the specialist health service have not prioritised helping you when you’ve told them about your stomach problems?

Yes

No

Don’t know

1. In all your encounters with the specialist health service, how often have you experienced that health professionals have not prioritised helping you when you’ve told them about your stomach problems?

| Never |  |
| --- | --- |
| Very rarely |  |
| Rarely |  |
| Often |  |
| Very often |  |
| Always |  |

1. Has this experience changed since the first time you told the specialist health service about your stomach problems?

Yes, I find that the specialist health service prioritises helping me more today than during the first consultation.

Yes, I find that the specialist health service prioritises helping me less today than during the first consultation.

No, I my experience of the specialist health service is the same today as during the first consultation.

| Never |  |
| --- | --- |
| Very rarely |  |
| Rarely |  |
| Often |  |
| Very often |  |
| Always |  |

1. How often do you find that the specialist health service meets you with courtesy and respect when you tell health professionals about your stomach problems?
2. To what extent do you agree or disagree with the following statement:

I find that health professionals in the specialist health service have sufficient expertise on irritable bowel syndrome.

| Totally disagree |  |
| --- | --- |
| Disagree |  |
| Slightly disagree |  |
| Neither disagree nor agree |  |
| Slightly agree |  |
| Agree |  |
| Totally agree |  |

1. To what extent do you agree or disagree with the following statement:

During my schooling and education, I have experienced that the educational institutions have shown little understanding and willingness to make adaptions for my stomach problems.

| Totally disagree |  |
| --- | --- |
| Disagree |  |
| Slightly disagree |  |
| Neither disagree nor agree |  |
| Slightly agree |  |
| Agree |  |
| Totally agree |  |
| I haven't told my school or educational institution about my stomach problems. |  |
| I haven't had any (major) problems with my irritable bowel during my schooling and education. |  |

1. To what extent do you agree or disagree with the following statement:

Over the course of my professional life, I have experienced that employers have shown little understanding and willingness to make adaptations for my stomach problems.

| Totally disagree |  |
| --- | --- |
| Disagree |  |
| Slightly disagree |  |
| Neither disagree nor agree |  |
| Slightly agree |  |
| Agree |  |
| Totally agree |  |
| I haven't told my employer about my stomach problems. |  |
| I haven't had any (major) problems with my irritable bowel during my professional life |  |
| I haven't worked in the labour market |  |

1. What treatment options and measures have you received from the public health service?

(you can select more than one option)

| Dietary guidance from a clinical nutritionist |  |
| --- | --- |
| Information about diet from a doctor/nurse |  |
| Hypnosis |  |
| Faecal transplant |  |
| Physiotherapy |  |
| Psychologist |  |
| Lactic acid bacteria (probiotics) |  |
| Prebiotics |  |
| Imodium |  |
| Enema/Laxatives |  |
| Non-prescription drugs for flatulence |  |
| Prescription painkillers |  |
| Non-prescription painkillers |  |
| Fibre supplements |  |
| Gastric acid neutralising drugs (reduces acid) |  |
| Gastric acid supplements (increases acid) |  |
| Medicinal cannabis |  |
| Constella |  |
| Resolor |  |
| Ondansetron |  |
| Eluxadoline: Truberzi/Viberzi |  |
| Visiblin |  |
| Other |  |

1. To what extent do you agree or disagree with the following statement:

I feel that the treatment offered by the public health service has helped me with my irritable bowel.

| Totally disagree |  |
| --- | --- |
| Disagree |  |
| Slightly disagree |  |
| Neither disagree nor agree |  |
| Slightly agree |  |
| Agree |  |
| Totally agree |  |

1. What types of treatment or measures have you tried out on your own without help from the public health service? (you can select more than one option)

| Dietary guidance |  |
| --- | --- |
| Diet |  |
| Hypnosis |  |
| Faecal transplant |  |
| Physiotherapy |  |
| Psychologist |  |
| Lactic acid bacteria (probiotics) |  |
| Prebiotics |  |
| Imodium |  |
| Enema/Laxatives |  |
| Non-prescription drugs for flatulence |  |
| Prescription painkillers |  |
| Non-prescription painkillers |  |
| Fibre supplements |  |
| Acupuncture |  |
| Colon cleansing |  |
| Digestive enzymes |  |
| Dietary supplements |  |
| Healing |  |
| Foot reflexology |  |
| Homeopathy |  |
| Fleawort |  |
| Functional medicine |  |
| Gastric acid neutralising drugs (reduces acid) |  |
| Gastric acid supplements (increases acid) |  |
| Medicinal cannabis |  |
| Visiblin |  |
| Other |  |

1. To what extent do you agree or disagree with the following statement:

I feel that the treatment I tried on my own has helped my irritable bowel.

| Totally disagree |  |
| --- | --- |
| Disagree |  |
| Slightly disagree |  |
| Neither disagree nor agree |  |
| Slightly agree |  |
| Agree |  |
| Totally agree |  |

1. At an estimate, how much money do you spend each year on treatment for your IBS that is not subsidised by the public health service?

| NOK 0 |  | |
| --- | --- | --- |
| NOK 1,000 |  | |
| NOK 2,000 |  | |
| NOK 3,000 |  | |
| NOK 4,000 |  | |
| NOK 5,000 |  | |
| NOK 6,000 |  | |
| NOK 7,000 |  | |
| NOK 8,000 |  | |
| NOK 9,000 |  | |
| NOK 10,000-14,000 |  | |
| NOK 15,000-19,000 |  | |
| NOK 20,000-24,000 |  | |
| NOK 25,000-29,000 |  | |
| NOK 30,000-34,000 |  | |
| NOK 35,000-39,000 |  | |
| NOK 40,000-44,000 |  | |
| NOK 45,000-49,000 |  | |
| NOK 50,000-54,000 |  | |
| NOK 55,000-59,000 |  | |
| NOK 60,000-64,000 |  | |
| NOK 65,000-69,000 |  | |
| NOK 70,000-74,000 |  | |
| NOK 75,000-79,000 |  | |
| NOK 80,000-84,000 |  | |
| NOK 85,000-89,000 |  | |
| NOK 90,000-94,000 |  | |
| NOK 95,000-99,000 |  | |
| NOK 100,000-109,000 |  | |
| NOK 110,000-119,000 |  | |
| NOK 120,000-129,000 |  | |
| NOK 130,000-139,000 |  |  |
| NOK 140,000-149,000 |  |  |
| NOK 150,000-159,000 |  |  |
| NOK 160,000-169,000 |  |  |
| NOK 170,000-179,000 |  |  |
| NOK 180,000-189,000 |  |  |
| NOK 190,000-199,000 |  |  |
| NOK 200,000-249,000 |  |  |
| NOK 250,000-299,000 |  |  |
| More than NOK 300 000 |  |  |

1. What measures or types of treatment do you feel have helped you the most with your irritable bowel?

The questionnaire used and patients’ answers were in Norwegian

**English translation**

Survey on Living with Irritable Bowel Syndrome (IBS)

2,727 Replies 13:34 Average completion time Closed Status

1. Sex

Male 297

Female 2,430

1. Age

| Under 13 | 5 |
| --- | --- |
| 13-17 years | 20 |
| 18-24 years | 166 |
| 25-29 years | 272 |
| 30-34 years | 300 |
| 35-39 years | 301 |
| 40-44 years | 321 |
| 45-49 years | 332 |
| 50-54 years | 344 |
| 55-59 years | 249 |
| 60-64 years | 188 |
| 65-69 years | 113 |
| 70-74 years | 86 |
| 75-79 years | 25 |
| 80-84 years | 2 |
| 85-89 years | 3 |
| Over 90 | 0 |

1. Place of abode

| Eastern Norway | 1,269 |
| --- | --- |
| Western Norway | 626 |
| Southern Norway | 168 |
| Central Norway | 369 |
| Northern Norway | 286 |
| I don't live in Norway | 9 |
|  |  |

1. Employment status

| Employed | 1,437 |
| --- | --- |
| Student | 217 |
| Incapacitated (100%) | 478 |
| Partially incapacitated | 166 |
| On sick leave (100%) | 100 |
| Partially on sick leave | 96 |
| Old age pensioner | 186 |
| School pupil | 38 |
| Homemaker | 18 |
| Receiving a work assessment allowance | 218 |
| Work placement | 26 |
| Unemployed | 15 |
| Laid off from work | 7 |
| Other | 57 |
|  |  |

1. Estimate the average number of days per year that you are/have been absent from work or education due to your irritable bowel.

| 0 days | 831 |
| --- | --- |
| 1-9 days | 802 |
| 10-19 days | 398 |
| 20-29 days | 185 |
| 30-39 days | 125 |
| 40-49 days | 49 |
| 50-59 days | 42 |
| 60-69 days | 57 |
| 70-79 days | 16 |
| 80-89 days | 21 |
| 3-4 months | 51 |
| 5-6 months | 35 |
| 7-8 months | 21 |
| 9-10 months | 9 |
| 11-12 months | 85 |
|  |  |

1. Are you incapacitated, on sick leave or receiving a work assessment allowance because of your irritable bowel?

Yes, IBS is the main reason I am incapacitated, on sick leave or receiving a work assessment allowance 170

Yes, IBS is one of the reasons why I am incapacitated, on sick leave or receiving a work assessment allowance 580

No 1,977

1. Have you been diagnosed with irritable bowel syndrome?

| Yes, it was diagnosed by my GP. | 805 |
| --- | --- |
| Yes, it was diagnosed by a gastrointestinal specialist. | 1,437 |
| Yes, it was diagnosed by another doctor (not a GP or gastrointestinal specialist). | 158 |
| No, I haven't received a diagnosis from the health service, I arrived at this conclusion on my own. | 205 |
| I don't have irritable bowel syndrome. | 26 |
| Other | 96 |

1. What irritable bowel symptoms do you have?

| Diarrhoea | 1,999 |
| --- | --- |
| Constipation | 1,703 |
| Stomach pain | 2,299 |
| Bloated stomach | 2,438 |
| Headache | 1,153 |
| Chronic fatigue | 1,721 |
| Other | 378 |
|  |  |

1. Approximately how long ago is it since you first developed symptoms of irritable bowel syndrome?

| Less than six months ago | 12 |
| --- | --- |
| 6-12 months | 24 |
| 1-2 years | 100 |
| 3-5 years | 343 |
| 6-10 years | 500 |
| 11-14 years | 394 |
| 15-19 years | 362 |
| 20-24 years | 277 |
| 25-29 years | 214 |
| Over 30 years ago | 475 |
|  |  |

1. If a doctor has diagnosed you with irritable bowel syndrome, approximately how long ago did you receive the diagnosis?

| Less than six months ago | 116 |
| --- | --- |
| 6-12 months | 124 |
| 1-2 years | 340 |
| 3-5 years | 645 |
| 6-10 years | 603 |
| 11-14 years | 237 |
| 15-19 years | 171 |
| 20-24 years | 110 |
| 25-29 years | 56 |
| Over 30 years ago | 50 |
| I haven't been diagnosed by a doctor | 249 |

1. To what extent does irritable bowel syndrome impair your quality of life?

Not at all 2

To a small degree 76

To some extent 938

To a great extent 1,194

To a very great extent 491

1. How often do you feel your quality of life is reduced because of your irritable bowel?

Never 24

Monthly 448

Weekly 1,058

Daily 940

Always 231

1. To what extent does irritable bowel syndrome impair your work/studies?

Not at all 171

To a small degree 396

To some extent 1,158

To a great extent 631

To a very great extent 345

1. To what extent does irritable bowel syndrome impair your social life?

Not at all 30

To a small degree 227

To some extent 1,062

To a great extent 938

To a very great extent 444

1. To what extent does irritable bowel syndrome impair your sex life?

Not at all 299

To a small degree 530

To some extent 1,041

To a great extent 574

To a very great extent 257

1. Are you often tired because of your irritable bowel?

Not at all 46

To a small degree 226

To some extent 831

To a great extent 1,017

To a very great extent 581

1. Do you feel repulsed by your irritable bowel?

Not at all 307

To a small degree 506

To some extent 877

To a great extent 609

To a very great extent 402

1. Approximately how long did it take before you visited a doctor after the onset of the first symptoms of irritable bowel syndrome?

| - A couple of days | 22 |
| --- | --- |
| - 1 week | 43 |
| 2-4 weeks | 143 |
| 1-3 months | 272 |
| 4-6 months | 211 |
| 6-12 months | 245 |
| 1-2 years | 324 |
| 2-3 years | 255 |
| 4-5 years | 160 |
| More than 5 years | 588 |
| I haven't seen a doctor because of this. | 80 |
| Don’t know | 358 |

1. Approximately how many times have you discussed your intestinal problems with your GP?

| Once | 85 |
| --- | --- |
| 2-3 times | 358 |
| 4-5 times | 474 |
| 6-9 times | 415 |
| 10-14 times | 375 |
| 15-19 times | 197 |
| 20-49 times | 278 |
| 50 times or more | 246 |
| I’ve never mentioned my intestinal problems to my GP. | 32 |
| Don’t know | 241 |

1. Approximately how many times did you discuss your intestinal problems with your GP before he/she sent you to a specialist?

| - Once | 267 |
| --- | --- |
| - 2-3 times | 564 |
| 4-5 times | 407 |
| 6-9 times | 239 |
| 10-14 times | 175 |
| 15-29 times | 87 |
| 30 times or more | 99 |
| I’ve never mentioned my intestinal problems to my GP. | 20 |
| My GP has never sent me to a specialist. | 604 |
| Don’t know | 239 |

1. Have you had to change your GP to be referred to a medical specialist?

Yes 451

No 2,250

1. How long did it take before you saw a specialist after your GP sent a referral?

| 1 day | 17 |
| --- | --- |
| 2-3 days | 23 |
| Approx. one week | 56 |
| Approx. 14 days | 119 |
| Approx. one month | 406 |
| 2-3 months | 566 |
| 4-6 months | 322 |
| 7-12 months | 116 |
| More than one year | 63 |
| More than two years | 29 |
| Don’t know | 335 |
| My GP has never sent me to a specialist. | 649 |

1. Have you seen a private specialist at your own expense for your intestinal problems?

Yes 743

No 1,958

1. Overall, approximately how long did it take from the time when you first saw a doctor until you were diagnosed with IBS?

| - Less than one week | 98 |
| --- | --- |
| - 1-3 weeks | 80 |
| Approx. one month | 142 |
| 2-3 months | 235 |
| 4-6 months | 218 |
| 7-12 months | 204 |
| 1-2 years | 305 |
| 3-4 years | 221 |
| 5 years or more | 615 |
| I haven't received a diagnosis from the health service, I arrived at this conclusion on my own. | 230 |
| I haven't spoken to a doctor about my intestinal problems. | 7 |
| Don’t know | 346 |

1. What tests and examinations were carried out when investigating irritable bowel syndrome? (you can select more than one option)

| Gastroscopy | 1,531 |
| --- | --- |
| - Colonoscopy | 1,808 |
| Blood tests | 1,961 |
| Stool samples | 2,103 |
| MRI | 342 |
| X-ray | 280 |
| Ultrasound | 532 |
| CT | 399 |
| Food allergy test | 917 |
| Food intolerance tests | 1,046 |
| Investigation for celiac disease | 1,168 |
| Don’t know | 50 |
| No tests have been taken | 125 |
| Other | 136 |

1. How satisfied are you with the help you have received from the GP service for your irritable bowel?

Very dissatisfied 290

Dissatisfied 428

Slightly dissatisfied 484

Neither dissatisfied nor satisfied 560

Somewhat satisfied 280

Satisfied 457

Very satisfied 202

1. Have you experienced your GP not believing you when you’ve told him/her about your stomach problems?

Yes 949

No 1,456

Don’t know 296

1. In all encounters with your GP, how often have you experienced that he/she has not believed you when you’ve told him/her about your stomach problems?

| - Never | 936 |
| --- | --- |
| - Very rarely | 426 |
| Rarely | 599 |
| Often | 490 |
| Very often | 197 |
| Always | 53 |

1. Has this experience changed since the first time you told your GP about your stomach problems?

Yes, I find that my GP believes me more now than he/or she did during the first consultation. 905

Yes, I find that my GP believes me less now than during the first consultation. 88

No, I find that my GP is still the same as during the first consultation. 1,630

I haven't spoken to my GP about my stomach problems. 78

1. Have you experienced that your GP hasn't prioritised helping you when you've told him/her about your stomach problems?

Yes 931

No 1,359

Don’t know 411

1. In all encounters with your GP, how often have you experienced that he/she has prioritised helping you when you’ve told him/her about your stomach problems?

| Never | 853 |
| --- | --- |
| Very rarely | 428 |
| Rarely | 615 |
| Often | 578 |
| Very often | 178 |
| Always | 49 |

1. Has this experience changed since the first time you told your GP about your stomach problems?

Yes, I find that my GP prioritises helping me more today than during the first consultation. 694

Yes, I find that my GP prioritises helping me less today than during the first consultation. 176

No, I find that my GP is still the same as during the first consultation. 1,757

I haven't spoken to my GP about my stomach problems. 74

1. How often do you find that your GP meets you with courtesy and respect when you tell him/her about your stomach problems?

| Never | 37 |
| --- | --- |
| Very rarely | 65 |
| Rarely | 307 |
| Often | 910 |
| Very often | 380 |
| Always | 1,002 |

1. To what extent do you agree or disagree with the following statement:

I find that the GP service has sufficient expertise on irritable bowel syndrome.

| - Totally disagree | 709 |
| --- | --- |
| - Disagree | 645 |
| Slightly disagree | 434 |
| Neither disagree nor agree | 427 |
| Slightly agree | 208 |
| Agree | 195 |
| Totally agree | 83 |

1. Have you been in contact with the specialist health service for your stomach problems?

Yes 1,653

No 1,048

1. How satisfied are you with the help you have received from the specialist health service for your irritable bowel?

Very dissatisfied 127

Dissatisfied 204

Slightly dissatisfied 226

Neither dissatisfied nor satisfied 305

Somewhat satisfied 275

Satisfied 373

Very satisfied 143

1. Have you experienced that health professionals in the specialist health service have not believed you when you’ve told them about your stomach problems?

Yes 387

No 1,111

Don’t know 155

1. In all your encounters with the specialist health service, how often have you experienced that health professionals have not believed you when you’ve told them about your stomach problems?

| - Never | 688 |
| --- | --- |
| - Very rarely | 278 |
| Rarely | 369 |
| Often | 228 |
| Very often | 71 |
| Always | 19 |

1. Has this experience changed since the first time you told health professionals in the specialist health service about your stomach problems?

Yes, I find that health professionals in the specialist health service believe me more today than during the first consultation. 320

Yes, I find that health professionals in the specialist health service believe me less today than during the first consultation. 64

No, I have the same experience of health professionals in the specialist health service today as during the first consultation. 1,269

1. Have you experienced that health professionals in the specialist health service have not prioritised helping you when you’ve told them about your stomach problems?

Yes 444

No 957

Don’t know 252

1. In all your encounters with the specialist health service, how often have you experienced that health professionals have not prioritised helping you when you’ve told them about your stomach problems?

| Never | 622 |
| --- | --- |
| Very rarely | 263 |
| Rarely | 412 |
| Often | 257 |
| Very often | 66 |
| Always | 33 |

1. Has this experience changed since the first time you told the specialist health service about your stomach problems?

Yes, I find that the specialist health service prioritises helping me more today than during the first consultation. 238

Yes, I find that the specialist health service prioritises helping me less today than during the first consultation. 106

No, I my experience of the specialist health service is the same today as during the first consultation. 1,309

| Never | 15 |
| --- | --- |
| Very rarely | 43 |
| Rarely | 186 |
| Often | 595 |
| Very often | 308 |
| Always | 506 |

1. How often do you find that the specialist health service meets you with courtesy and respect when you tell health professionals about your stomach problems?

1. To what extent do you agree or disagree with the following statement:

I find that health professionals in the specialist health service have sufficient expertise on irritable bowel syndrome.

| Totally disagree | 165 |
| --- | --- |
| Disagree | 212 |
| Slightly disagree | 257 |
| Neither disagree nor agree | 315 |
| Slightly agree | 206 |
| Agree | 376 |
| Totally agree | 122 |

1. To what extent do you agree or disagree with the following statement:

During my schooling and education, I have experienced that the educational institutions have shown little understanding and willingness to make adaptions for my stomach problems.

| Totally disagree | 55 |
| --- | --- |
| Disagree | 65 |
| Slightly disagree | 58 |
| Neither disagree nor agree | 274 |
| Slightly agree | 131 |
| Agree | 160 |
| Totally agree | 181 |
| I haven't told my school or educational institution about my stomach problems. | 978 |
| I haven't had any (major) problems with my irritable bowel during my schooling and education. | 799 |

1. To what extent do you agree or disagree with the following statement:

Over the course of my professional life, I have experienced that employers have shown little understanding and willingness to make adaptations for my stomach problems.

| Totally disagree | 144 |
| --- | --- |
| Disagree | 229 |
| Slightly disagree | 122 |
| Neither disagree nor agree | 319 |
| Slightly agree | 262 |
| Agree | 239 |
| Totally agree | 227 |
| I haven't told my employer about my stomach problems. | 884 |
| I haven't had any (major) problems with my irritable bowel during my professional life | 195 |
| I haven't worked in the labour market | 80 |

1. What treatment options and measures have you received from the public health service?

(you can select more than one option)

| Dietary guidance from a clinical nutritionist | 778 |
| --- | --- |
| Information about diet from a doctor/nurse | 1,014 |
| Hypnosis | 10 |
| Faecal transplant | 77 |
| Physiotherapy | 140 |
| Psychologist | 265 |
| Lactic acid bacteria (probiotics) | 524 |
| Prebiotics | 154 |
| Imodium | 644 |
| Enema/Laxatives | 404 |
| Non-prescription drugs for flatulence | 543 |
| Prescription painkillers | 441 |
| Non-prescription painkillers | 336 |
| Fibre supplements | 457 |
| Gastric acid neutralising drugs (reduces acid) | 839 |
| Gastric acid supplements (increases acid) | 36 |
| Medicinal cannabis | 2 |
| Constella | 256 |
| Resolor | 55 |
| Ondansetron | 12 |
| Eluxadoline: Truberzi/Viberzi | 12 |
| Visiblin | 897 |
| Other | 528 |

1. To what extent do you agree or disagree with the following statement:

I feel that the treatment offered by the public health service has helped me with my irritable bowel.

| Totally disagree | 843 |
| --- | --- |
| Disagree | 595 |
| Slightly disagree | 260 |
| Neither disagree nor agree | 431 |
| Slightly agree | 328 |
| Agree | 187 |
| Totally agree | 57 |

1. What types of treatment or measures have you tried out on your own without help from the public health service? (you can select more than one option)

| Dietary guidance | 1,231 |
| --- | --- |
| Diet | 2,034 |
| Hypnosis | 62 |
| Faecal transplant | 114 |
| Physiotherapy | 291 |
| Psychologist | 343 |
| Lactic acid bacteria (probiotics) | 1,363 |
| Prebiotics | 649 |
| Imodium | 748 |
| Enema/Laxatives | 538 |
| Non-prescription drugs for flatulence | 995 |
| Prescription painkillers | 279 |
| Non-prescription painkillers | 765 |
| Fibre supplements | 753 |
| Acupuncture | 328 |
| Colon cleansing | 165 |
| Digestive enzymes | 506 |
| Dietary supplements | 1,146 |
| Healing | 163 |
| Foot reflexology | 200 |
| Homeopathy | 226 |
| Fleawort | 394 |
| Functional medicine | 129 |
| Gastric acid neutralising drugs (reduces acid) | 629 |
| Gastric acid supplements (increases acid) | 162 |
| Medicinal cannabis | 25 |
| Visiblin | 600 |
| Other | 282 |

1. To what extent do you agree or disagree with the following statement:

I feel that the treatment I tried on my own has helped my irritable bowel.

| Totally disagree | 236 |
| --- | --- |
| Disagree | 292 |
| Slightly disagree | 197 |
| Neither disagree nor agree | 517 |
| Slightly agree | 768 |
| Agree | 470 |
| Totally agree | 221 |

1. At an estimate, how much money do you spend each year on treatment for your IBS that is not subsidised by the public health service?

| NOK 0 | 235 | |
| --- | --- | --- |
| NOK 1,000 | 332 | |
| NOK 2,000 | 307 | |
| NOK 3,000 | 299 | |
| NOK 4,000 | 214 | |
| NOK 5,000 | 316 | |
| NOK 6,000 | 134 | |
| NOK 7,000 | 97 | |
| NOK 8,000 | 57 | |
| NOK 9,000 | 49 | |
| NOK 10,000-14,000 | 359 | |
| NOK 15,000-19,000 | 78 | |
| NOK 20,000-24,000 | 85 | |
| NOK 25,000-29,000 | 37 | |
| NOK 30,000-34,000 | 18 | |
| NOK 35,000-39,000 | 17 | |
| NOK 40,000-44,000 | 14 | |
| NOK 45,000-49,000 | 4 | |
| NOK 50,000-54,000 | 16 | |
| NOK 55,000-59,000 | 5 | |
| NOK 60,000-64,000 | 1 | |
| NOK 65,000-69,000 | 1 | |
| NOK 70,000-74,000 | 3 | |
| NOK 75,000-79,000 | 0 | |
| NOK 80,000-84,000 | 3 | |
| NOK 85,000-89,000 | 1 | |
| NOK 90,000-94,000 | 0 | |
| NOK 95,000-99,000 | 4 | |
| NOK 100,000-109,000 | 6 | |
| NOK 110,000-119,000 | 1 | |
| NOK 120,000-129,000 | 2 | |
| NOK 130,000-139,000 | 0 |  |
| NOK 140,000-149,000 | 2 |  |
| NOK 150,000-159,000 | 0 |  |
| NOK 160,000-169,000 | 1 |  |
| NOK 170,000-179,000 | 0 |  |
| NOK 180,000-189,000 | 0 |  |
| NOK 190,000-199,000 | 1 |  |
| NOK 200,000-249,000 | 2 |  |
| NOK 250,000-299,000 | 0 |  |
| More than NOK 300 000 | 0 |  |

1. What measures or types of treatment do you feel have helped you the most with your irritable bowel?

2701 answers **“none”**
